# Nasal Continuous Positive Airway Pressure Inhibits Gastroesophageal Reflux in Newborn Lambs

**DOI:** 10.1371/journal.pone.0107736

**Published:** 2014-09-16

**Authors:** Djamal Djeddi, Danny Cantin, Nathalie Samson, Jean-Paul Praud

**Affiliations:** 1 Neonatal Respiratory Research Unit, Departments of Pediatrics and Physiology, Université de Sherbrooke, Sherbrooke, Quebec, Canada; 2 Pediatric Department, Amiens University Medical Center, Amiens, France; University of Cincinnati, United States of America

## Abstract

**Background:**

Using esophageal pHmetry, nasal CPAP (nCPAP) has been shown to decrease acid gastroesophageal reflux (GER) in adult humans. Although both GER (mainly non-acid) and nCPAP use are very frequent in newborns, the effect of nCPAP on GER in early life is unknown. Having recently shown that the newborn lamb is a unique model for studying neonatal GER, our main objective was to assess the effect of nCPAP on GER in newborn lambs.

**Methods:**

Eight newborn lambs, aged 2–3 days, were studied. Continuous esophageal pH-Impedance monitoring and polysomnography were performed for six hours during both spontaneous breathing and nCPAP application at 6 cmH_2_O (nCPAP6), in a randomized order. Results were compared in the two experimental conditions, as well as without CPAP during the following 6 hours.

**Results:**

i) nCPAP_6_ virtually abolished GER [mean ±SD reflux number for 6 h = 9.1±8.6 without nCPAP_6_ vs. 0.6±1 with nCPAP_6_, P<0.05]; ii) GER number was also reduced during the 6 h-period following nCPAP6 application (18±16 without nCPAP_6_ vs. 7±8.1 with nCPAP_6_, P<0.05); iii) nCPAP_6_ decreased the depth and duration of lower esophageal sphincter relaxation.

**Conclusions:**

nCPAP inhibits GER in the newborn lamb. Further clinical studies using different levels of nasal CPAP are needed to confirm this result in human infants.

## Introduction

There has been a recent unprecedented development of nasal respiratory support for neonates in an attempt to prevent bronchopulmonary dysplasia and cerebral palsy, both of which have been associated with the use and duration of endotracheal intubation and ventilation in neonates [1], [2]. Hence, nasal continuous positive airway pressure (nCPAP) and various nasal intermittent positive pressure ventilation modalities are currently being used and tested for their relative advantages [3].

The use of nasal respiratory support, including nCPAP, has been associated with the passage of air into the esophagus along with previous published reports of gastric dilation and feeding intolerance in neonates [3]. Such reports raise the possibility of increased gastro-esophageal refluxes (GER) with nCPAP application in neonates. Conversely, nCPAP has been shown to decrease pathological acid GER in adults with [4] and without [5] obstructive sleep apnea (OSA).

In the past few years, we have used our newborn ovine model to show the absence of effects of nCPAP on nutritive swallowing [6] and, more specifically, on esophagodeglutition [7]. In addition, we have established the newborn lamb as a unique model to study neonatal GER [8]. In keeping with our research program on nCPAP and gastroesophageal function in the neonatal period, the aim of the present study was to test the hypothesis that nCPAP decreases GER number in our neonatal ovine model, using Multichannel Intraluminal Impedance-pH monitoring (MII-pH).

## Materials and Methods

The eight lambs involved in the study were born at term by spontaneous vaginal delivery. They were aged between 2 to 3 days and weighed 3.2±0.5 Kg on the experimental day. The protocol design also included an assessment of the effects of nCPAP on nutritive esophagodeglutition at the end of the experimentation, which has been the subject of a previous publication [7]. All lambs were cared for without their mother in our animal quarters, due to specific needs regarding bottle-feeding familiarization. The animals moved freely in a Plexiglas chamber and could bottle-feed with reconstituted ewe milk *ad libitum*. The chamber (1.2 m^3^; in agreement with recommendations by the Canadian Council for Animal Care) was placed in a temperature-controlled room at 26°C.

### nCPAP and recording equipment

Lambs were instrumented immediately prior to recordings. Nasal continuous positive airway pressure was induced using the Infant Flow system (Cardinal Health, Dublin, OH) with heated humidified air. A custom-built nasal mask [9] was installed on the lamb's muzzle to deliver nCPAP, in such a way that the lambs could feed from a bottle freely [6].

Lambs underwent an esophageal MII-pH (MMS, Enschede, Holland). The MII-pH catheter (diameter  = 2 mm, Unisensor, Portsmouth, USA) was inserted trans-nasally and its position confirmed by X-ray [8]. External lamb instrumentation included i) an electrocardiogram for monitoring heart rate; ii) a pulse oximeter probe (Masimo Radical, Irvine, CA) attached at the base of the tail for monitoring oxygen hemoglobin saturation; iii) thoracic and abdominal bands of respiratory inductive plethysmography for monitoring of respiratory rate.

The above physiological signals were wirelessly transmitted and continuously recorded on a PC using AcqKnowledge (4.1, Biopac Systems, Montreal, Canada) and MMS (8.2) softwares. The entire recording period was also filmed using a webcam.

In addition, pressure measurements were performed using an esophageal manometry catheter with solid-state transducers specifically designed for lambs (Gaeltec Inc, Scotland). The catheter is equipped with three high-fidelity pressure sensors and one 4-cm long circumferential sphincter transducer (sphincterometer) positioned at 4-cm intervals from the catheter tip. The manometry catheter was connected to the same PC as above to allow synchronized data acquisition and analysis of esophageal manometry. No oro- or naso-gastric tube was placed during the experiments beside the impedance catheter, which did not pass through the lower esophageal sphincter.

### Design of the study

The study was performed without sedation. The MII-pH probe was left in place for 48 h to allow for comparison of two different conditions, namely no nCPAP (control) and nCPAP at 6cmH_2_O (nCPAP_6_) in randomized order, performed at the same time of day on both days. The level of nCPAP was chosen on the basis of what is usually reported in clinical practice [10]. The lambs were placed in a sling with loose restraints during the six hours of nCPAP application (or control condition). Four hours after beginning of the recording, they were offered 75 ml of ewe milk at ambient temperature (26°C), using the same bottle and teat for all lambs.

At the end of the six-hour recording, each lamb underwent an esophageal manometry, while still installed in the sling, in both the control and nCPAP_6_ condition. Following calibration, the catheter was introduced orally into the stomach to measure gastric baseline pressure (P_GB_), then pulled out into the esophagus using the rapid pull-through method, such that the lower esophageal sphincter (LES) as well as proximal, mid and distal esophagus were facing a pressure sensor. Thereafter, one-mL boluses of water at ambient temperature were offered, at least 30 seconds after any motor activity. Measurements included end expiratory esophageal resting pressures (proximal, mid and distal P_es_ and P_LES_), as well as transdiaphragmatic pressure (P_TD_ = P_GB_ – distal P_es_) and LES barrier pressure (P_b_ =  end-expiratory P_LES_ - P_GB_) [11]. Peristaltic wave amplitude and duration in the proximal, mid and distal esophagus were averaged during three completely transmitted swallows. The percentage LES relaxation, an index of the depth of LES relaxation, was calculated as the ratio of the difference between the lowest P_LES_ during LES relaxation and basal P_LES_ over basal P_LES_. Spontaneous swallows were used for calculation if they met the same timing criterion [12].

Following manometry, lambs were returned to the Plexiglas chamber, where they could move freely and bottle-feed with reconstituted ewe milk *ad libitum*, while the esophageal MII-pH recording was continued until the next morning.

The study was approved by the ethics committee for animal care and experimentation of the Université de Sherbrooke (protocol # 283–11).

### Data analysis

Statistical analyses were performed on raw data for all variables to compare no CPAP conditions to nCPAP_6_. Values are expressed as mean (standard deviation). Given the non-Gaussian distribution for all studied variables, the Wilcoxon signed rank test was used for all comparisons (SPSS version 20, Chicago, IL, USA). A P value <0.05 was considered statistically significant. In addition, given the relatively small number of studied lambs (related both to the complexity of the ovine model and ethical constraints), it was decided to give full consideration in the discussion to the presence of a significant trend, defined as P<0.1.

## Results

Mean lamb body temperature was 39.6±0.4°C. Baseline oxygen hemoglobin saturation was identical in both control and nCPAP_6_ conditions at 95±2%. Mean heart rates in control and nCPAP_6_ conditions were respectively 179±7 and 202±12 bpm (P>0.1) while mean respiratory rates in control and nCPAP_6_ conditions were respectively 58±22 and 50±14 min^−1^. All MII-pH and pressure variables were successfully studied in both conditions (Table 1 and 2).

**Table 1:** pH-Impedancemetry results for no CPAP and nCPAP_6_ conditions during six hours in newborn lambs.

	No CPAP (n = 8)	nCPAP_6_ (n = 8)	*P* Value
***pH monitoring results***			
Reflux index, % of recording duration with pH<4	0	0	NS
Mean lower esophageal pH	5.5±0.1	5.8±0.1	NS
***Impedance-detected refluxes***			
Number of refluxes	9.1±8.6	0.6±1	0.02
Weakly acid refluxes, %	87.5±30.5	89±19	0.07
Acid refluxes, %	0±0	0±0	NS
Alkaline refluxes, %	12.5±30.5	11±19	NS
Extension to z1, %	32±38	0±0	0.04
BEI, s	116±158	10±19	0.06
Median bolus clearance time, s	2.5±4.5	0.3±0.1	0.06

Data are expressed as mean ±SD*;* nCPAP_6_ =  nasal CPAP+6 cmH_2_O; z1 =  impedance channel 1 (proximal esophagus); BEI =  Bolus Exposure Index; NS =  non-significant.

### nCPAP effects on gastroesophageal reflux

**Figure 1:**
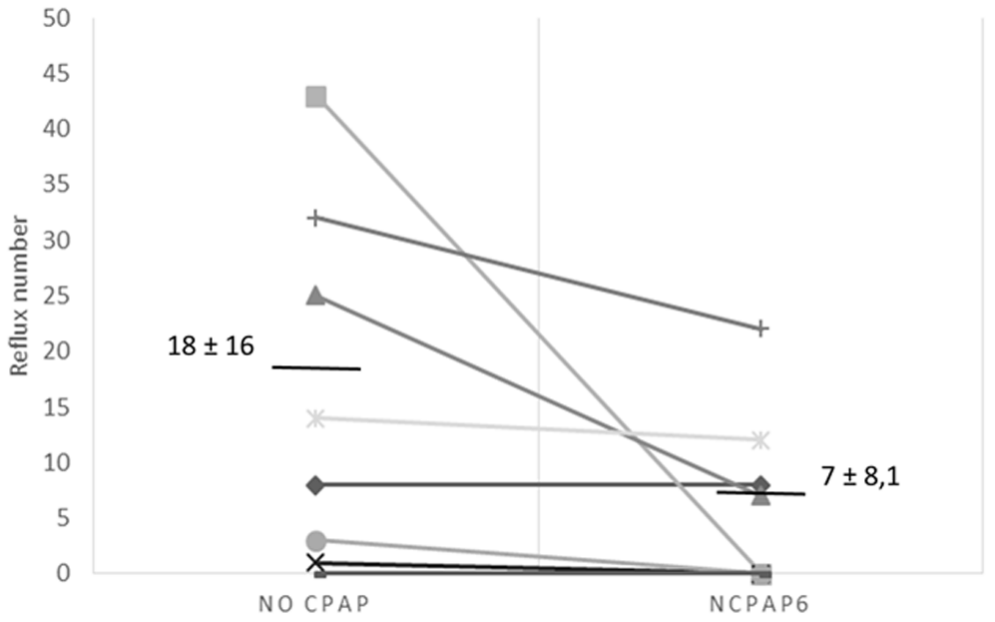
Number of reflux events for no CPAP and nCPAP_6_ conditions in newborn lambs during the six-hour recording period while in the sling. Abbreviations: nCPAP_6_ =  nasal CPAP+6 cmH_2_O.

Nasal CPAP_6_ virtually abolished GER [mean ±SD reflux number for 6 hours  = 9.1±8.6 without nCPAP_6_ *vs*. 0.6±1 with nCPAP_6_, P<0.05]. Moreover, no proximal reflux was observed under nCPAP_6_, while the median bolus clearance time tended to decrease (Figure 1 and Table 1).

**Figure 2:**
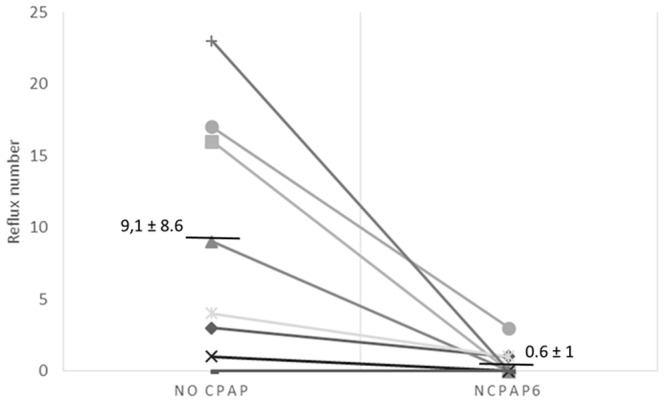
Number of reflux events during the 6 h-period following recording in the sling with either nCPAP_6_ application (right) or control condition (left). The overall greater number in refluxes compared to figure 1 is related to the lamb being now allowed to move and feed at will in the Plexiglas chamber. Abbreviations: nCPAP_6_ =  nasal CPAP+6 cmH_2_O.

The number of GER and the median bolus clearance time were also reduced during the six-hour period following nCPAP_6_ application compared to control condition, respectively from 18±16 to 7±8.1 (Figure 2) and from 0.9±0.3 s to 0.3±0.1 s, P<0.05. This reduction in GER number did not vary during the six-hour period following nCPAP_6_ (1±1.2 GER during the first hour post nCPAP_6_ *vs.* 0.5±0.7 GER during the sixth hour post nCPAP_6_). Meanwhile, the proximal reflux number was not significantly decreased during the six-hour period following nCPAP_6_ application compared to the six-hour post control period (respectively 2±0.8 vs. 5±2.3, P = 0.8). Finally, the apparent decrease in the bolus exposure index during the six-hour post nCPAP6 period compared to the six-hour post control period was not significant [0.1±0.1 s vs. 0.5±0.2 s, P>0.1]. Finally, while the bolus exposure index was decreased during the six-hour post nCPAP_6_ period compared to the six-hour post control period, this decrease was not significant [0.1±0.1 s *vs.* 0.5±0.2 s, P>0.1].

### nCPAP effects on manometry

No effects of nCPAP_6_ were observed for resting proximal, mid and distal P_es_, as well as for basal P_GB_ (Table 2). Similarly, peristaltic wave amplitude and duration were not affected by nCPAP_6_ application at any of the esophageal levels studied (Table 3).

**Table 2:** Manometry results for no CPAP and nCPAP_6_ conditions in newborn lambs.

	*No CPAP (n = 7)*	*nCPAP_6_ (n = 7)*
P_GB_, mmHg	3.5±2	3±1.5
P_LES_, mmHg	12.5±5.5	14.5±5
P_b_, mmHg	9.7±0.9	6.4±1
P_TD_, mmHg	6.3±3.2	8.4±3.3
Proximal P_es_ (basal), mmHg	−1.5±0.5	1.5±1.5
Mid P_es_ (basal), mmHg	−1±2	1.5±2
Distal P_es_ (basal), mmHg	−6.5±2.5	−3±0.5

Data are expressed as mean ±SD; P>0.1 for all comparisons. nCPAP_6_ =  nasal CPAP+6 cmH_2_O; P_GB_ =  basal intragastric pressure; P_LES_ =  resting end-expiratory lower esophageal sphincter pressure; P_b_ =  barrier pressure; P_TD_ =  transdiaphragmatic pressure; P_es_ =  resting esophageal pressure. All pressures are referenced to atmospheric pressure.

**Table 3:** Changes in swallow-induced esophageal peristaltic wave amplitude and duration in newborn lambs.

	*No CPAP (n = 7)*	*nCPAP_6_ (n = 7)*
**Peristaltic wave duration, s**		
*Proximal esophagus*	0.5±0.1	0.5±0.1
*Mid esophagus*	0.5±0.1	0.5±0.1
*Distal esophagus*	0.5±0.1	0.5±0.1
**Peristaltic wave amplitude, mmHg**		
*Proximal esophagus*	157.5±20	160±17.5
*Mid esophagus*	165.5±39.5	172.5±32.5
*Distal esophagus*	130.5±31.5	142±18

Data are expressed as mean ±SD; P>0.1 for all comparisons. nCPAP_6_ =  nasal CPAP+6 cmH_2_O. All pressures are referenced to atmospheric pressure.

A high-pressure zone characteristic of a LES was present at the esogastric junction in all lambs (Table 2). Nasal CPAP_6_ application did not significantly alter baseline P_LES_, P_TD_ or P_b_. However, nCPAP_6_ decreased both the percentage (control 130.9±39.6 vs. nCPAP_6_ 46.9±12.9%, P = 0.046) and duration (control 1.4±1.1 and nCPAP_6_ 0.7±1.1 s, P = 0.06) of LES relaxation during primary peristalsis.

## Discussion

The key finding of the present study is that the number of GER assessed by MII-pH in newborn lambs was dramatically decreased by nCPAP at 6 cmH_2_0. The latter was associated with a decrease in both the duration and depth of swallow-induced relaxation of the lower esophageal sphincter, which suggests that nCPAP may enhance the barrier function of the lower esophageal sphincter against GER. The uniqueness of these results resides in the fact that they were obtained in the neonatal period together with pH and impedance monitoring. The latter permitted to include weakly-acid refluxes and proximal refluxes. In addition, we demonstrate that the inhibiting effects of CPAP on GER persist for hours after cessation of nCPAP

### nCPAP effects on gastroesophageal refluxes

Our results are of significant importance given the prevalence of GER and GER disease in the neonatal period [13] as well as to the current extensive use of nCPAP in neonates [3]. The inhibiting effect of nCPAP upon acid refluxes has previously been reported in adult humans, including questionnaire-based studies [14] or esophageal pH monitoring in subjects suffering from OSA, nocturnal GER disease or aperistaltic esophagus [4],[5],[15]–[18]. In addition, two studies have shown a similar effect of nCPAP on acid GER in healthy adults using esophageal manometry and/or pH monitoring [5], [11]. The availability of our unique ovine neonatal model for the study of GER and esophagodeglutition [7], [8] allows for the first time to provide data in the neonatal period. The present study clearly shows that the inhibiting effect of nCPAP on GER is also present in early life and in the absence of esophageal disease. Moreover, the use of MII-pH provides additional new data. First, nCPAP also reduces weakly-acid refluxes, which are frequently involved in GER disease in early life [19]. Secondly, results show that nCPAP decreases the number of proximal refluxes, which are especially prominent in infants and can be responsible for cardiorespiratory inhibition *via* the laryngeal chemoreflexes, a group of reflexes triggered by the contact between a liquid and the laryngeal mucosa [20]. Of note, the laryngeal chemoreflexes have been involved in some cases of apnea of prematurity, apparent life-threatening events in infancy and sudden infant death syndrome [20]–[22]. Of particular interest, we previously showed that nCPAP significantly decreases the cardiorespiratory inhibition observed during experimentally-induced laryngeal chemoreflexes in preterm lambs [23]. The present results fittingly complement the findings of the former study, showing that nCPAP not only decreases the number of GER reaching the proximal esophagus (and thus potentially the laryngeal mucosa), but also prevents the potentially dramatic cardiorespiratory consequences of laryngeal chemoreflexes in newborns. Taken together, results from these 2 studies suggest a potential role for nCPAP in severe GER disease resistant to conventional medical treatments, including when cardiorespiratory consequences are present in infants. This suggestion is in agreement and extends a previous proposal in adults with nocturnal GER disease due to acid refluxes [5].

### Potential mechanisms explaining nCPAP effects

Studies in adults initially suggested that the inhibiting effect of nCPAP on GER was related to a passive rise in intraesophageal pressure and/or a LES constriction reflex [4], [5], [24]. However, more recent results suggest nCPAP acts by decreasing the duration and depth of LES relaxations [11]. Our present study brings further credence to this latter hypothesis. Indeed, although resting P_b_ did not increase significantly with application of nCPAP, the duration and depth of swallow-induced LES relaxation both decreased.

According to Mittal et al. (2005), in the presence of normal anatomy, the pressure at the gastroesophageal junction is the most important determinant of anti-reflux barrier strength and any factor that affects gastroesophageal junction pressure would in turn affect the flow across this junction [25]. Extrapolation from our results on swallow-induced LES relaxation suggests that nCPAP_6_ is such a factor. Definitive evidence would come from the demonstration that nCPAP decreases the occurrence of transient LES relaxations, which constitute the main mechanism responsible for GER, including in newborns [26].

The putative explanation behind the decrease in LES relaxation with nCPAP is not straightforward. Owing to the previous recognition that transient LES relaxations are associated with longitudinal esophageal muscle contraction, [27]–[29] Shepherd et al. (2007) hypothesized that the downward displacement of the diaphragm and mediastinal contents brought about by nCPAP expanded the esophagus longitudinally, thus increasing the preload of the longitudinal esophageal muscle [11]. This however remains to be tested.

The persistent beneficial effect of nCPAP_6_ upon GER after its discontinuation is intriguing. It extends previous similar, albeit unexplained, observations on acid GER after application of nCPAP during one week in adults with OSA [17]. Whether this memory effect is related to a type of striated (longitudinal esophageal muscle) or smooth (LES) muscle plasticity, as described for airway smooth muscle [30] and/or to certain persistent alterations in the activity of the numerous neurohumoral substances controlling LES contraction/relaxation is unknown [31]. Nevertheless, such finding may potentially be of high clinical relevance. Indeed, this persistent beneficial effect of nCPAP on GER suggests that nocturnal nCPAP may be a useful treatment in selected cases of severe GER disease in which conventional medical therapy has failed or involving life-threatening complications. Nasal CPAP could thereby represent a valuable alternative to Nissen fundoplication, especially when GER is expected to resolve with age.

### Potential limitations of the study

Transposition of the present results from newborn lambs to human infants must be made with caution. Anatomical differences with regard to the extent of striated muscle along the esophagus (upper third only in humans *vs.* entire esophagus in the sheep) are manifest [32]. However, there are clear similarities between the two species, including i) a well-established LES, ii) the fact that the preruminant lamb is monogastric in the first week of life [33] and iii) the presence of spontaneous GER, with characteristics close to human infants [8]. While our study was only limited to a few hours, similar results in adult humans with OSA suggest that the effect can persist for at least one week [17]. While it is not known whether CPAP levels other than 6 cmH_2_0 would impact GER differently, the level chosen herein is of common usage in newborns [3]. In addition, further studies are needed to assess whether nasal IPPV, which is increasingly used in newborns, similarly inhibits GER.

## Conclusions

Nasal CPAP efficiently inhibits non-acid GER, which are most prominent in early life. Of significant importance for infants is the observation that this effect includes an inhibition of proximal GER. Moreover, the fact that the beneficial effect of nCPAP on GER persists several hours after nCPAP discontinuation offers the perspective of using nocturnal nCPAP for treating selected cases of severe GERD.
