# Correction: Nasal Continuous Positive Airway Pressure Inhibits Gastroesophageal Reflux in Newborn Lambs

**DOI:** 10.1371/journal.pone.0115157

**Published:** 2014-12-04

**Authors:** The PLOS ONE Staff

The images for Figures 1 and 2 are incorrectly switched. The image that appears as Figure 1 should be Figure 2, and the image that appears as Figure 2 should be Figure 1. The figure legends appear in the correct order. Please view the correct images and legends here.

**Figure 1:**
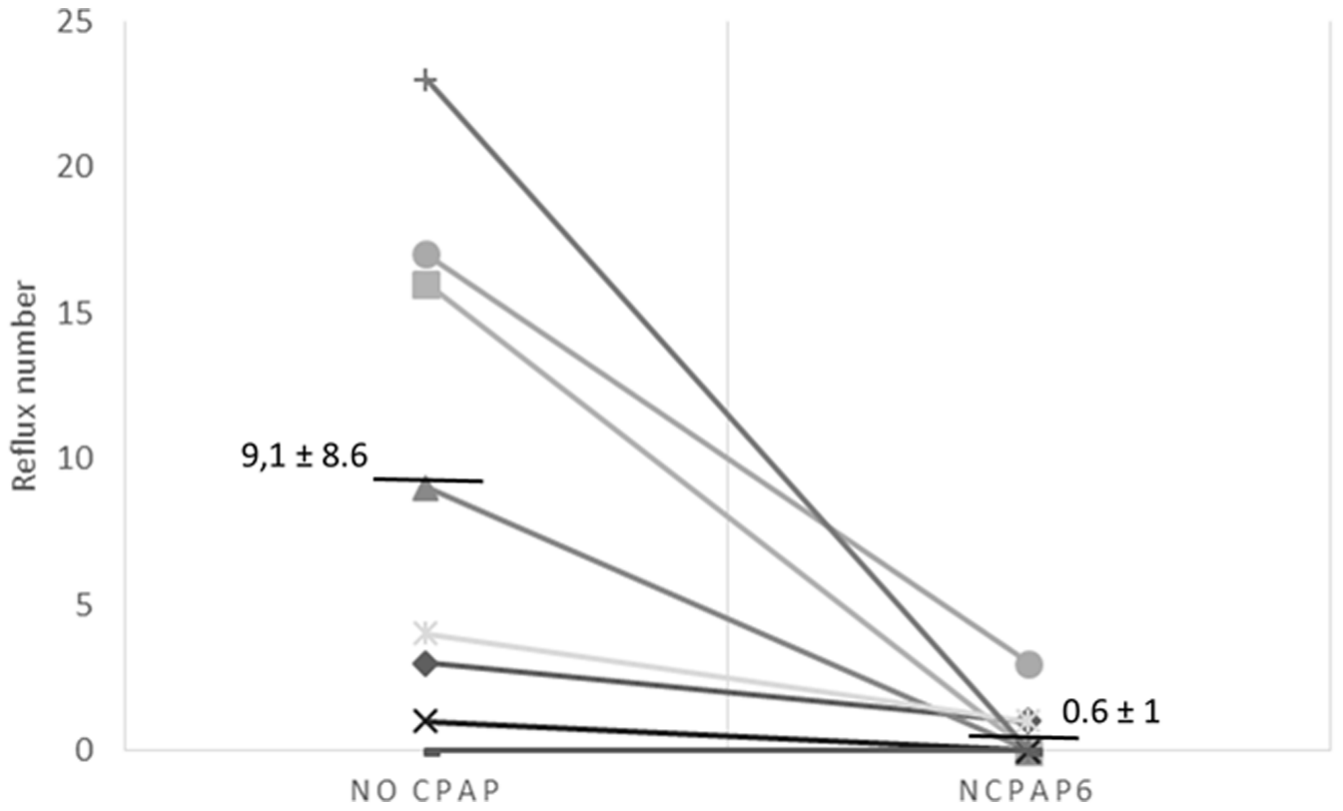
Number of reflux events for no CPAP and nCPAP_6_ conditions in newborn lambs during the six-hour recording period while in the sling. Abbreviations: nCPAP_6_  =  nasal CPAP+6 cmH_2_O.

**Figure 2:**
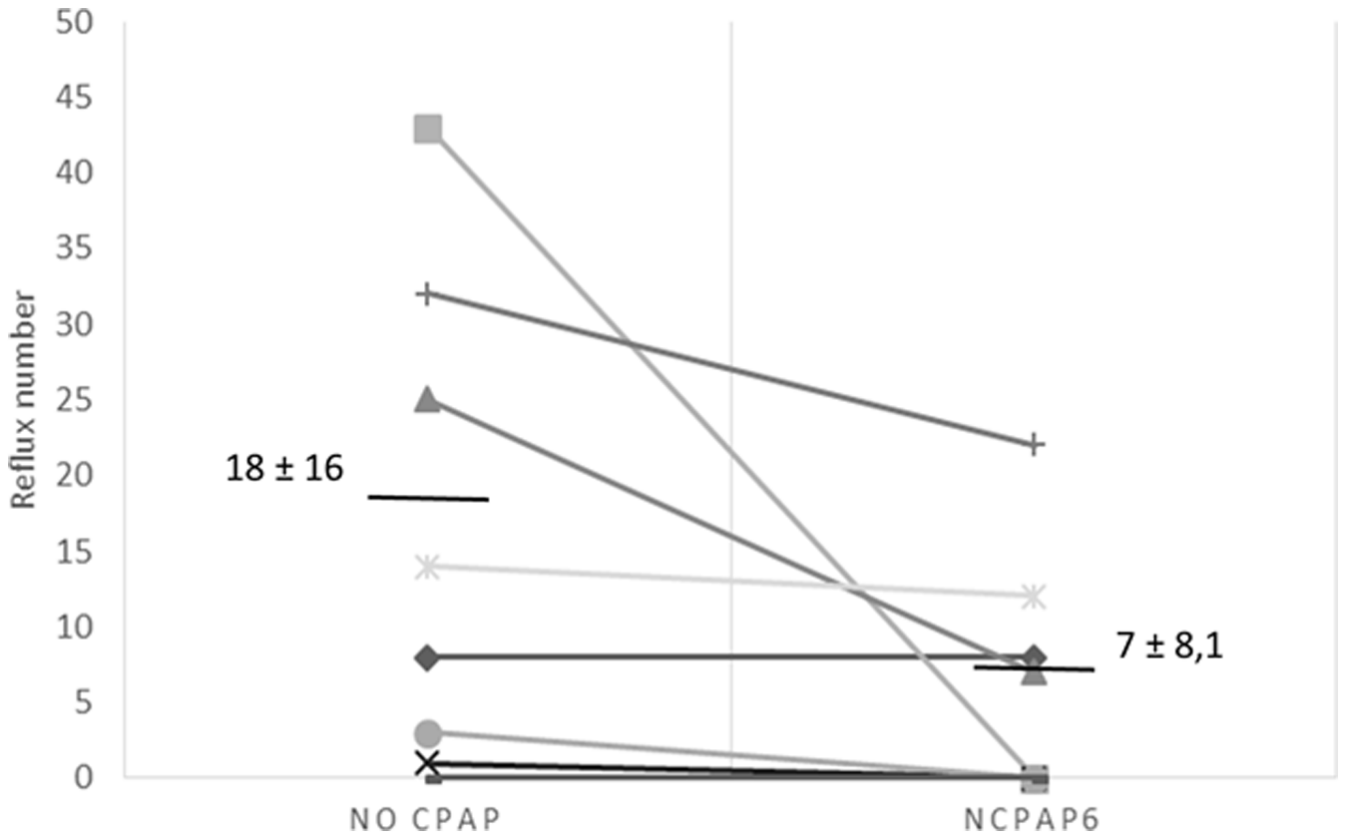
Number of reflux events during the 6 h-period following recording in the sling with either nCPAP_6_ application (right) or control condition (left). The overall greater number in refluxes compared to figure 1 is related to the lamb being now allowed to move and feed at will in the Plexiglas chamber. Abbreviations: nCPAP_6_ =  nasal CPAP+6 cmH_2_O.
